# Modern Sociology

**Published:** 1902-01-25

**Authors:** 


### MODERN SOCIOLOGY.

### WOMEN'S RESTAURANTS.

### III.—SOME SCHEMES.

VARIOUS schemes have from time to time been trotted out for providing restaurants the prices of which shall be within the limits demanded by the very poor. Sir Edward Sullivan's penny dinners for school children in West India Docks, and Mrs. Warner Snoad's public kitchen in Battersea, are instances of this type of enterprise, but the inauguration of public kitchens on a large and self-supporting basis is so far a thing of the future. At the present time Mr. D. Tallerman is inaugurating a scheme for providing London with a "family pie" at 2d. a head, the idea being to utilise the 3,000 odd existing bakeries and to eliminate the question of rent. It is perhaps hardly fair to make the unqualified statement that what has been done on the Continent can be equally well done in England. For one thing the tastes of the people are different—the mess of pottage, the mingling of bitter and sweet, on which the German working class has been reared, would be most unacceptable to the palate of the British workwoman. Climatic conditions vary, and, moreover, the average woman in this country does not value vegetable and cereal food as her Continental neighbour does.

The fact remains, however, that while we have hardly reached even the experimental stage, people's kitchens have been flourishing on the Continent for many years. In 1892 the founders of the Lodgings Society of Lyons started a Food Society, having for its object the opening up of popular restaurants. Each gave 2,000 francs towards defraying the initial expenses of buying furniture, etc., and the Rhône Savings Bank gave 6,000 francs towards the building of the restaurant, which was opened in the Brotteaux quarter. Since the first year the profits have been at the rate of 6½ per cent. From the beginning the receipts allowed of a ready payment for the stocks and furniture, so that the associates did not need to make the payment, and the enterprise may be said to have been worked without capital. In 1895 another restaurant was started in the Guillotière quarter. Both continue to prosper. In 1900, 11,793 meals were served on an average each day to 3,063 customers, of which the average cost of production per person was 44 centimes. The whole receipts amounted to 488,292fr. 75c. As in other restaurants of this kind, the meals are paid for by tokens valued from 10 to 20 centimes, which the people purchase when passing through the turnstile. There is, in addition, a "service token," which costs 15 centimes, and for this the consumer is admitted to a special room, where there is a special attendant, and where a serviette is provided. About a hundred diners make use of this provision.

Monsieur Joseph Cernesson, of Sens (Yonne), has been kind enough to furnish us with a specimen tariff, as follows:—

| | Centimes. |
|---|---|
| Pain (environ 150 grammes) ... ... ... ... | 0·05 |
| Viande ou poisson (environ 140 grammes) ... | 0·20 |
| Légumes ou pâtes alimentaires (une forte assiette) ... ... ... ... ... ... | 0·10 |
| Soupe grasse ou maigre (80 centilitres) ... | 0·10 |
| Dessert varié suivant la saison... ... ... | 0·10 |
| Café (noir) ... ... ... ... ... ... | 0·10 |
| Café-cognac ... ... ... ... ... ... | 0·20 |

The last item is a concession to the popular taste, and not altogether one to be recommended.

Other restaurants, "co-opératifs ou populaires," exist in Paris, Grenoble, Bourges, Geneva, and Milan. Of the last, Mons. Cernesson says: "C'est tout à fait remarquable, mais c'est surtout un hôtel."

Vienna and Berlin have also had flourishing Volks Küchen for many years past; they are established in the poorer quarters of the city, and the food is prepared and sold at the lowest possible price consistent with the principle of self-support. In Vienna there are now 11 of these kitchens, where thousands of people breakfast, dine, and sup daily. The founder is Dr. Joseph Kühn, who collected the necessary capital, in the shape of public subscriptions, and invested funds for the initial expenses. His principle was and is "No profit, and voluntary help in the management"; in other respects the kitchens are conducted on strict business lines, and it appears to be due to the efforts of one individual that they are so strikingly successful.

The Berliner Volks-Küchen-Verein has also carried on public kitchens with conspicuous success. Herr Herrmann Abraham is the President of the Verein; he originally (that is to say, some five or six years ago) opened a small dining-room where students and workmen of small means could get an inexpensive but well-cooked meal for a sum corresponding to our threepence, the portions being one penny each. One morning he met a group of hungry children outside, who said they were waiting for "leavings." On inquiring into their parents' circumstances, he discovered that the need for cheap meals was greater than he had supposed; the hungry children proved an inspiration to him, and the now flourishing kitchens of Berlin give free meals to very needy children. "There shall be no more hungry children in Berlin," he resolved, and it is a significant fact that, low as the prices are, every one of these restaurants has proved self-supporting "from the moment the doors were opened." Comparing prices in Berlin with those in London wholesale houses, it has been found that on an average they are much the same, some things being a trifle dearer, but most of them cheaper, here than in Berlin. Mr. C. Herman Senn, who has devoted a good deal of attention to the study of how the cooking is done in the homes of the English poor, and instances the wretched *Batterie de cuisine* (often consisting of the mere frying-pan), and the want of knowledge on the part of the majority of the people, has come to the conclusion that two things are necessary—(1) more instruction in cooking and the choice of food; and (2) supply kitchens in every parish, where good, wholesome and well-prepared food should be cooked and sold at the lowest possible prices. The County Councils, the Universal Cookery and Food Association (of which Mr. Senn is Hon. Director), and other agencies already give free cooking demonstrations; and Miss Clara James, to whose work among girls we have already referred, welcomes our suggestion that lessons on the choice and relative value of foods would be most useful in girls' clubs.

The objection that public supply kitchens (whether for distributing dinners or supplying food on the premises) would destroy home life seems upon examination to be a worthless one; and in Berlin, at any rate, it has been found that the public kitchens have raised the standard of private cookery. The mere saving of fuel and labour would be immense, when one remembers that, as Frau Morgenstern (Berlin) points out, 300 to 1,000 families can be served from one kitchen, with one fire, and a staff of from four to twelve persons, whereas, in private houses, 300 to 1,000 women are occupied with as many fires, cooking inferior meals at a much greater cost. "I never pays more than 1d. or 1½d. for my dinner," said a poor woman when offered a good meal for 3d.; and it is for such women, whether mothers of families obliged to go out to work, or singly fighting the battle of life on a miserable wage, that public kitchens are so urgently wanted. Could there be a more suitable opening for municipal enterprise than the supply of people's kitchens? And if, in such a scheme, it were found possible to supply a limited amount of good beer (as is the case in some of the Continental kitchens), say half a pint and no more with each meal, so much the better.

